# Supplementary material for: EVATOM: an optical, label-free, machine learning assisted embryo health assessment tool
Source: Commun Biol. 2024 Mar 5;7:268. doi: 10.1038/s42003-024-05960-w (PMC10915136; doi:10.1038/s42003-024-05960-w)
Supplement: Supplementary file 1 — Supplementary Information [file 42003_2024_5960_MOESM1_ESM.pdf]

Supplementary information for

EVATOM: an optical, label-free, machine learning assisted embryo health assessment tool

Neha Goswami[1,2,#], Nicola Winston[3], Wonho Choi[4], Nastasia Z. E. Lai[4], Rachel B. Arcanjo[4,7], Xi Chen[2,8], Nahil Sobh[5], Romana A. Nowak[4,#], Mark A. Anastasio[1,2,6,#] and Gabriel Popescu[1,2,6,9]

[1]Department of Bioengineering, University of Illinois Urbana-Champaign, Urbana, IL 61801, USA

[2]Beckman Institute of Advanced Science and Technology, University of Illinois Urbana-Champaign, IL 61801, USA

[3]Division of Reproductive Endocrinology and Infertility, Department of Obstetrics and Gynecology, University of Illinois at Chicago College of Medicine, Chicago, IL 60612, USA

[4]Department of Animal Sciences, University of Illinois Urbana-Champaign, Urbana, IL 61801, USA

[5]NCSA Center for Artificial Intelligence Innovation, University of Illinois Urbana-Champaign, Urbana, IL 61801, USA

[6]Department of Electrical and Computer Engineering, University of Illinois Urbana-Champaign, Urbana, IL 61801, USA

[7]Present address: Department of Animal Science, University of California, Davis, CA 95616, USA

[8]Present address: School of Applied and Engineering Physics, Cornell University, Ithaca, NY 14850, USA

[9]Deceased: Gabriel Popescu

#nehag4@illinois.edu, #ranowak@illinois.edu, #maa@illinois.edu

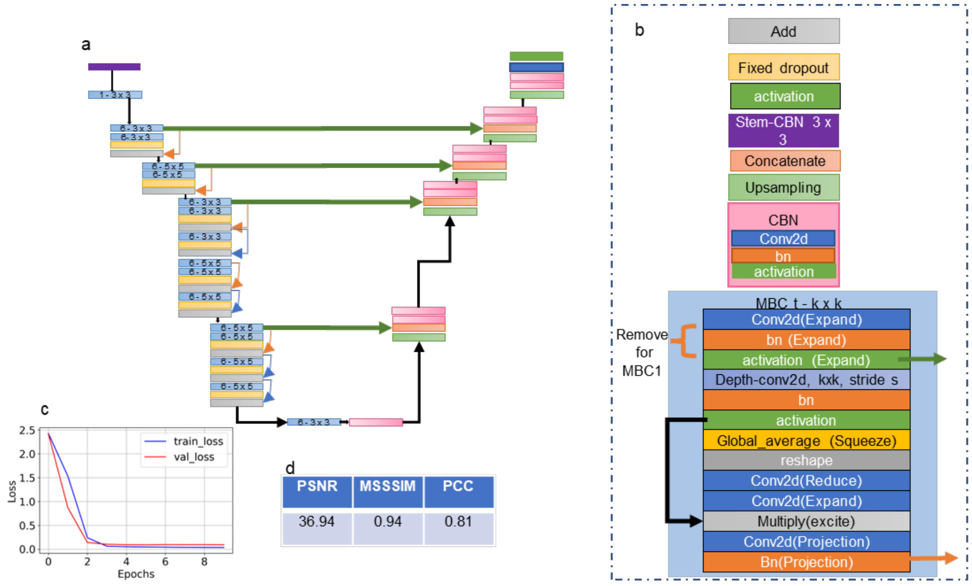

PSNR	MSSSIM	PCC
36.94	0.94	0.81

Supplementary Figure 1. Nucleus prediction model architecture a. Model architecture of Efficient Net-B0 UNet used for nuclei detection. In this model, a pretrained Efficient Net B0 block replaces the encoder part of the UNet. b. Block descriptions of a, c. Loss curve for the model, d. Performance metrics on the test dataset.

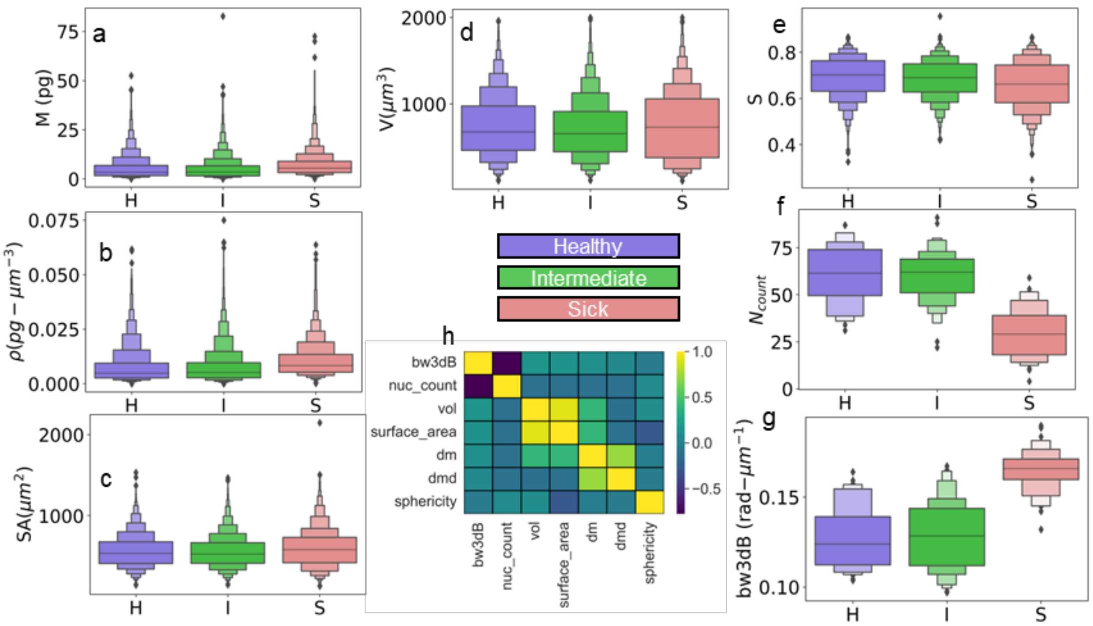

Supplementary Figure 2. Features: Boxenplots for a. Dry mass, b. Dry mass density, c. Surface area, d. Volume, e. Sphericity, f. Nucleus count, and g. bw3dB for healthy (blue), intermediate (green) and sick (red) embryos. h. Correlation heat map for all features. Statistics is presented in Supplementary Table 1.

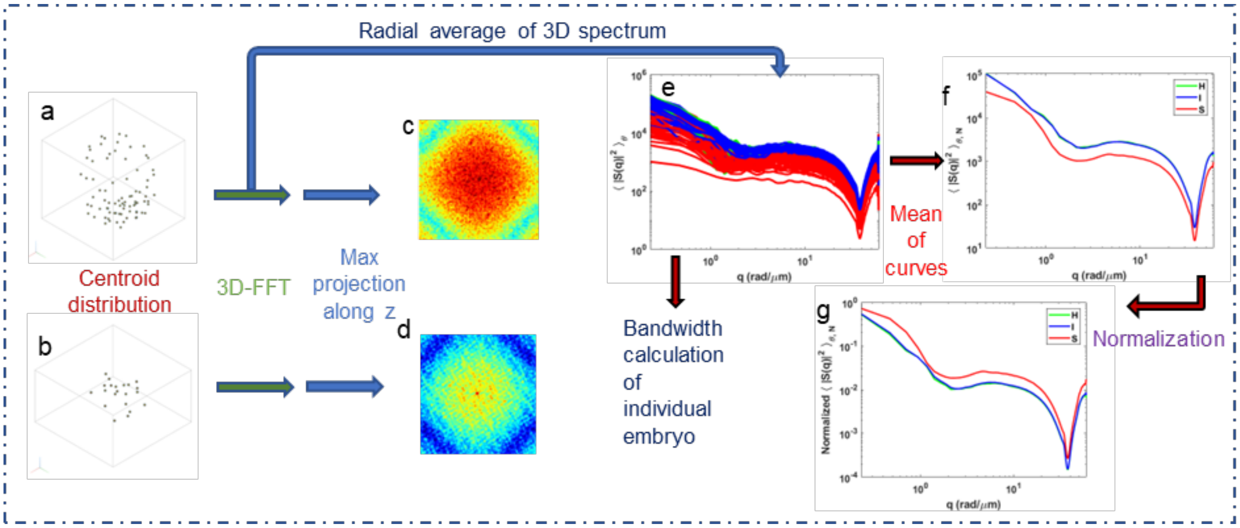

Supplementary Figure 3. Scattering amplitude bandwidth calculation: a, b. Centroid distribution from the 3d segmentation, where a unit sphere is placed at each centroid for healthy/intermediate (a) and sick embryo (b). Centroid distribution is then 3D Fourier transformed into $\mathbf{q}$-domain. c and d show the maximum projections of scattering amplitude spectrums along the $\mathbf{k}_z$ direction for visualization purposes only. Note that the calculation of associated bandwidths considers whole 3D volume information and not max projections. e. Radially averaged power spectral density of scattering amplitude associated with all 152 embryos, f. Mean of curves per class, g. Normalized mean radially averaged power spectral density for each class. Red curves denote sick class, green curves denote healthy class and blue curves denote intermediate class.

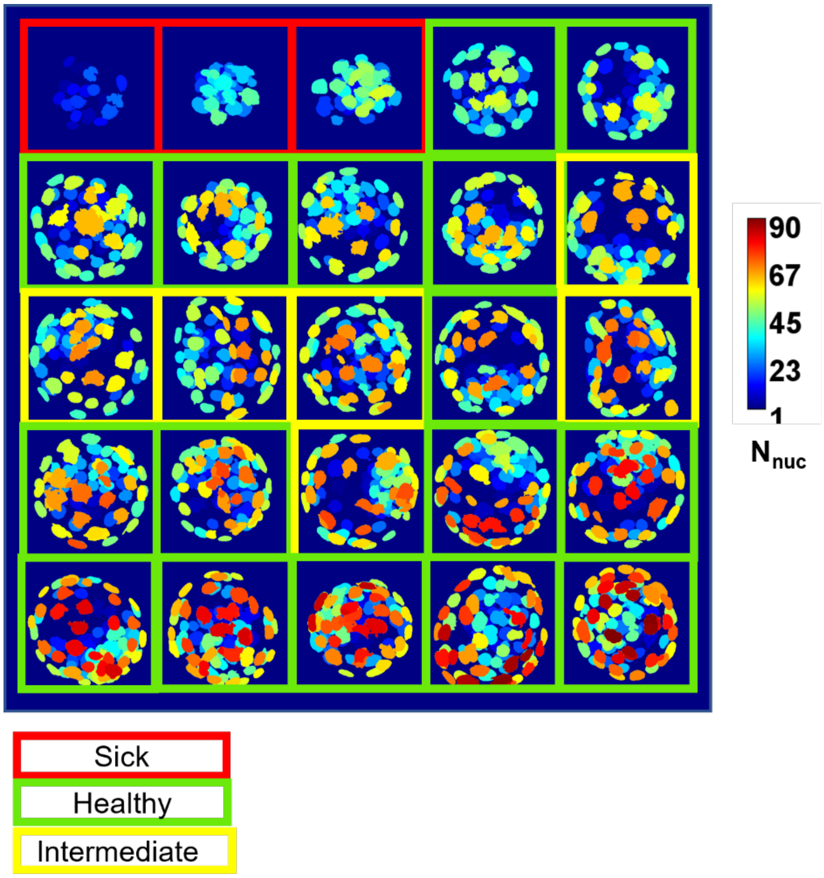

Supplementary Figure 4. Nucleus count distribution for a batch of embryos, with embryos in red, green, and yellow boxes belonging to classes Sick, Healthy, and Intermediate, respectively. Colorbar shows the nucleus count distribution.

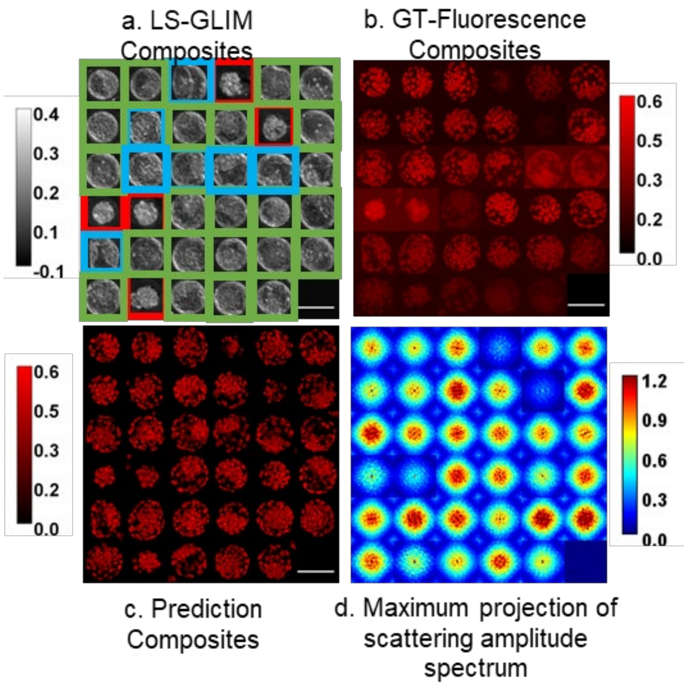

Supplementary Figure 5. Example of differences in scattering amplitude spectrum for healthy/intermediate and sick classes. a. LS-GLIM maximum phase projections along z-axis with red boxes showing sick embryos, green boxes showing healthy embryos and blue boxes showing intermediate embryos. Colorbar represents phase distribution b. Ground truth maximum intensity projections of nuclei fluorescence image corresponding to embryos in a, Colorbar represents normalized intensity c. Maximum intensity projections of nuclei model predictions, Colorbar represents normalized intensity d. maximum intensity projections of scattering amplitude spectrum for embryos in a. Colorbar represents spectrum intensity. Scalebar is 100 µm for a, b and c.

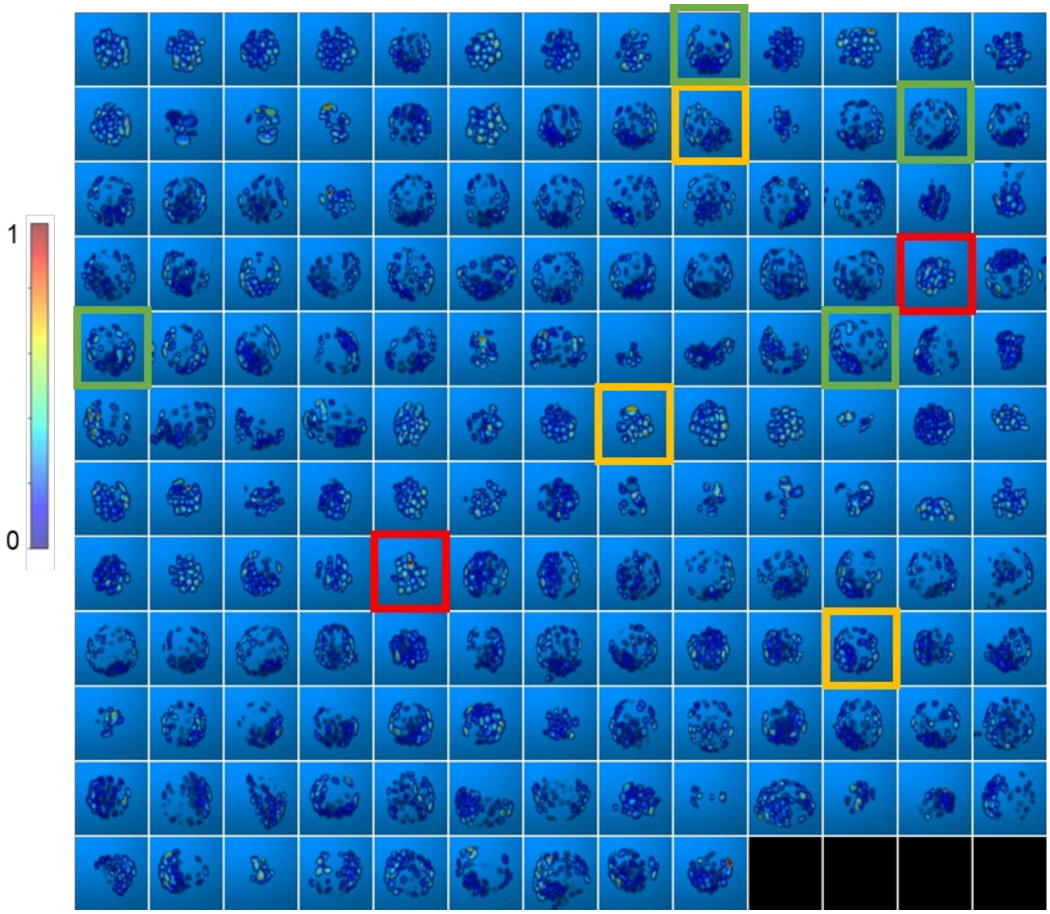

Supplementary Figure 6. Normalized mean nuclear dry-mass density distribution. 3D reconstructions of mean nuclear dry mass density map for 152 embryos with sick (enclosed in red box), intermediate (enclosed in orange box), and healthy (enclosed in green box) embryos shown in main text Figure 4. The colorbar shows the normalized mean nuclear dry mass density.

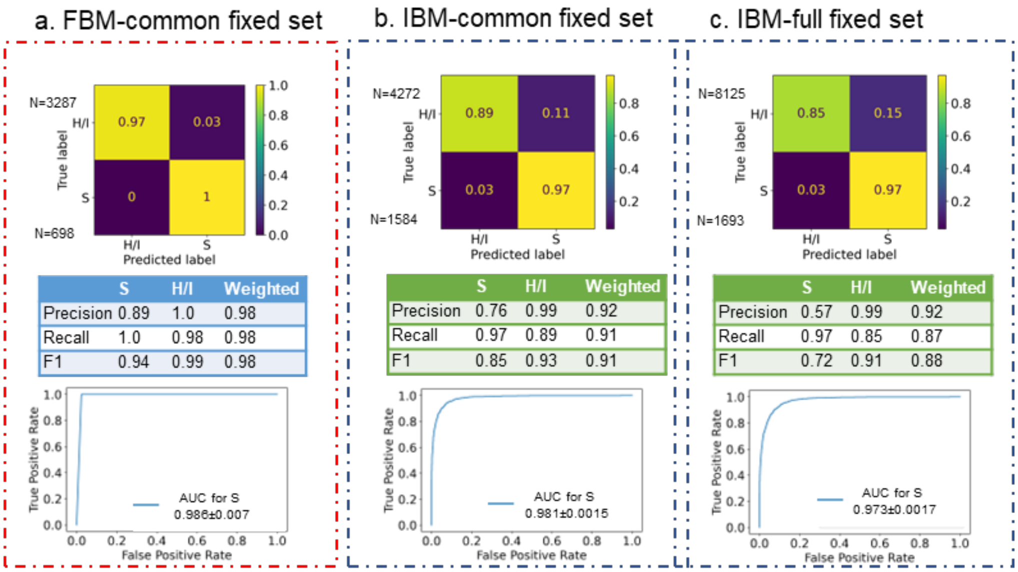

Supplementary Figure 7. Health grading baseline model performance: confusion matrix, performance metrics, ROC curve for in-distribution test set. a. FBM (nuclei level-72 embryos), b. IBM (z-slice level-72 embryos), c. IBM (z-slice level-122 embryos). AUC values are indicated with +/- standard error of the mean.

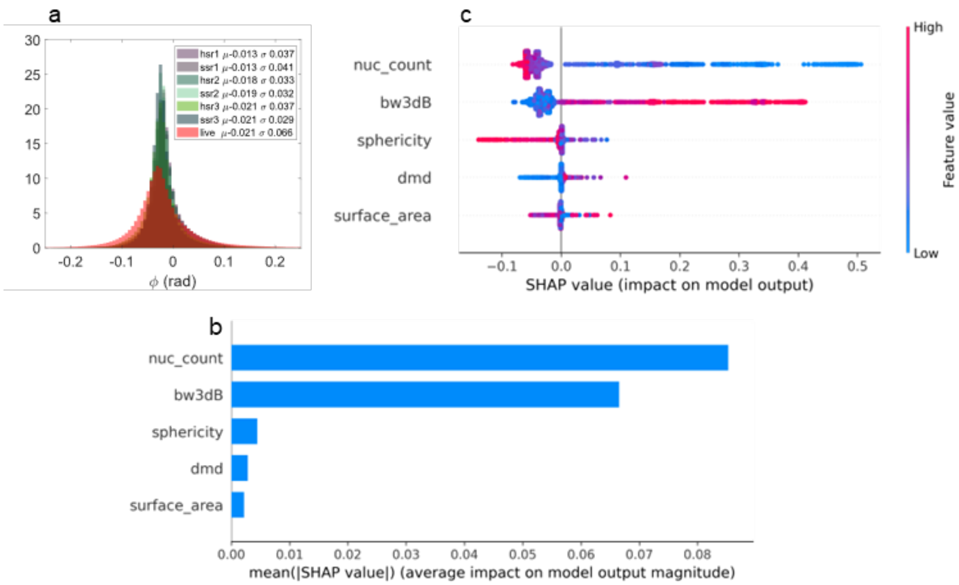

Supplementary Figure 8. a. Phase distribution histogram between in-distribution datasets (hsr1, ss1, hsr2, ssr2, hsr3 and ssr3) versus out-of-distribution dataset (live). b. SHAP feature importance plot for FBM, c. SHAP summary plot for FBM.

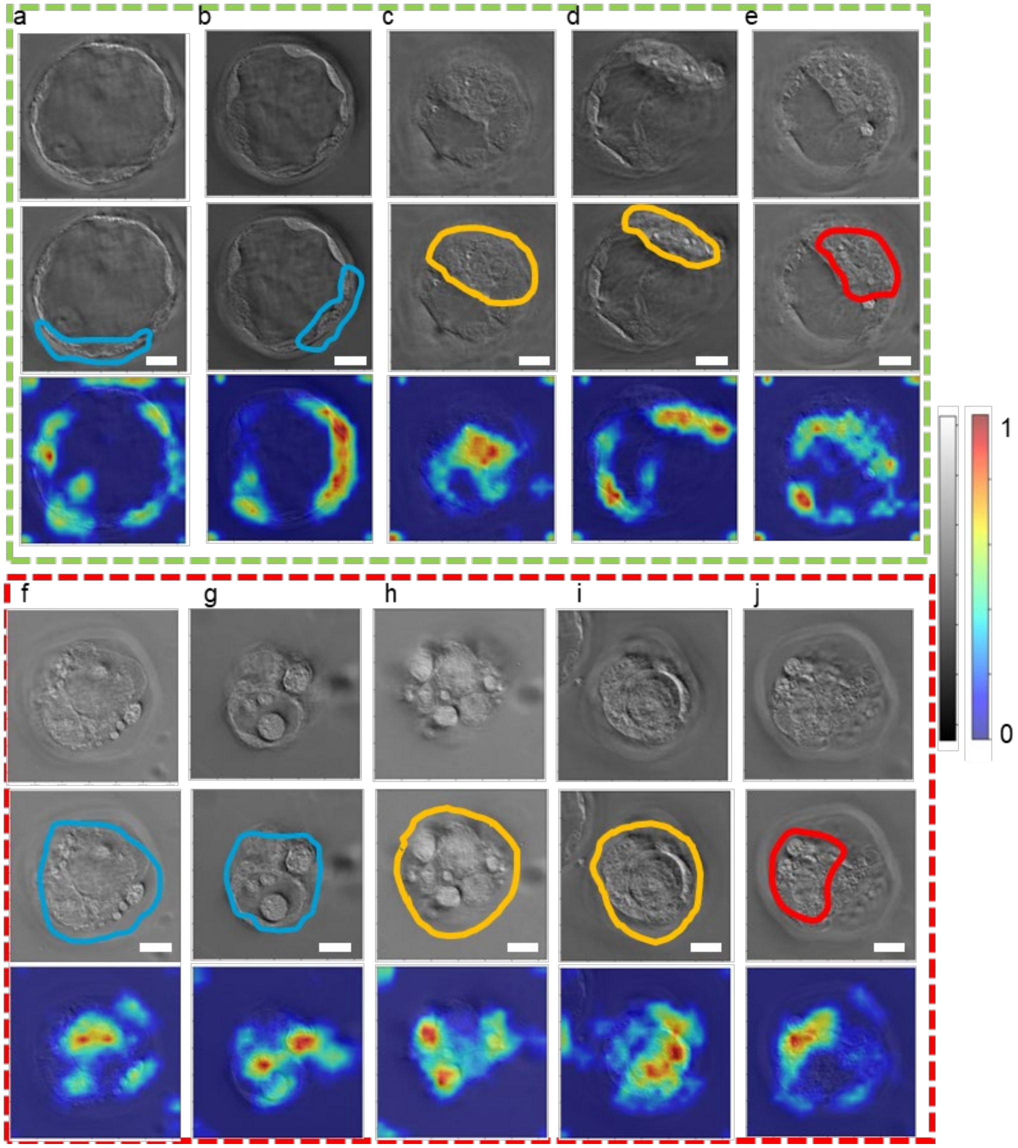

Supplementary Figure 9. Grad-CAM interpretability test of IBM: for healthy (a-e) and sick (f-j) embryos for successful predictions: The top row in each panel is a random z-slice of different embryos with the corresponding overlayed Grad-CAM image in the bottom row. Results are compared with blind markings from three experts (shown with different colors) in the middle row. Colorbar for GLIM images shows normalized phase maps and the color bar for Grad-CAM outputs shows the importance of the regions for correct prediction with red being the highest. Scalebars are shown as white rectangles on the lower right corner of images in the middle row and represent 20 µm.

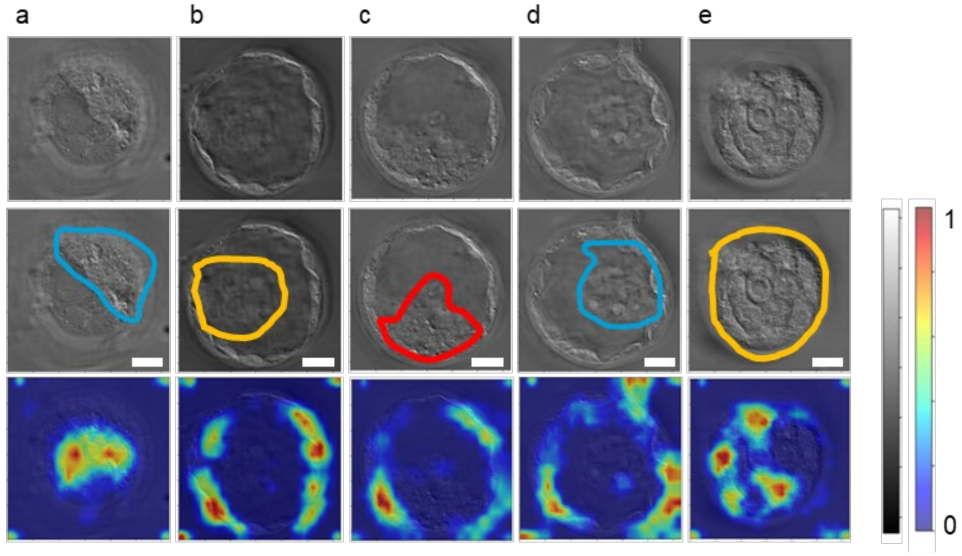

Supplementary Figure 10. Grad-CAM interpretability test of IBM: for healthy (a-e) embryos for successful predictions where expert decisions do not fully agree: The top row in each panel is a random z-slice of different embryos with the corresponding overlayed Grad-CAM image in the bottom row. Results are compared with blind markings from three experts (shown with different colors) in the middle row. Colorbar for GLIM images show normalized phase maps and the colorbar for Grad-CAM outputs shows the importance of the regions for correct prediction with red being the highest. Scalebars are shown as white rectangles on the lower right corner of images in the middle row and represent 20 µm.

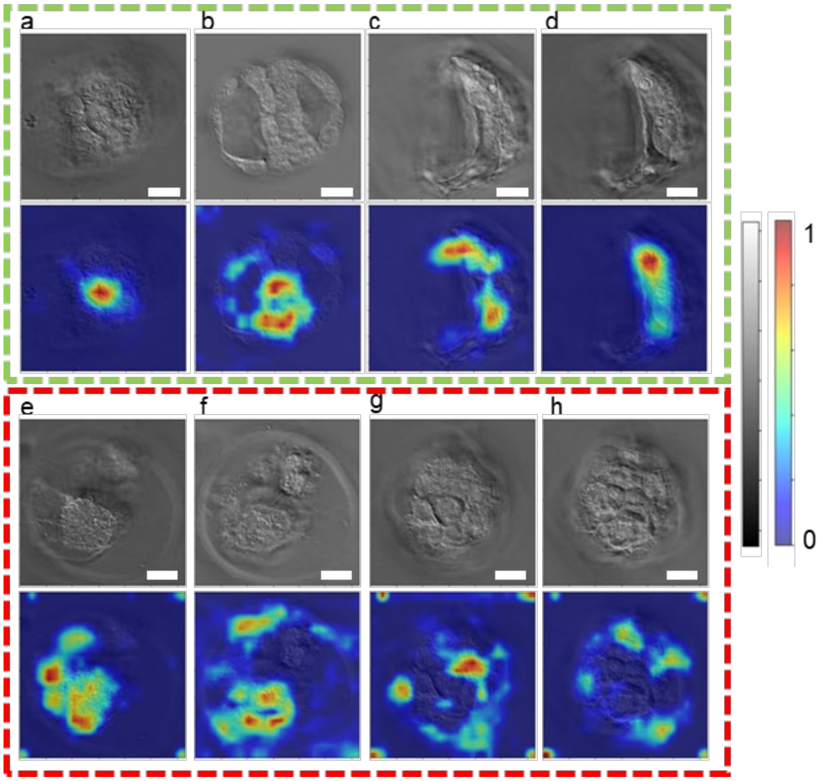

Supplementary Figure 11. Grad-CAM interpretability test of IBM: for wrong predictions: (a-d). Healthy z-slices predicted as sick, (e-h). sick z-slices predicted as healthy. Pair of slices per embryo are shown such that a and b belong to one embryo, c and d belong to another embryo, e and f belong to another embryo, and g and h belong to another embryo. Colorbar for GLIM images show normalized phase maps and the colorbar for Grad-CAM outputs shows the importance of the regions for prediction with red being the highest. Scalebars are shown as white rectangles on the lower right corner of images in the top row of each subfigure and represent 20 μm.

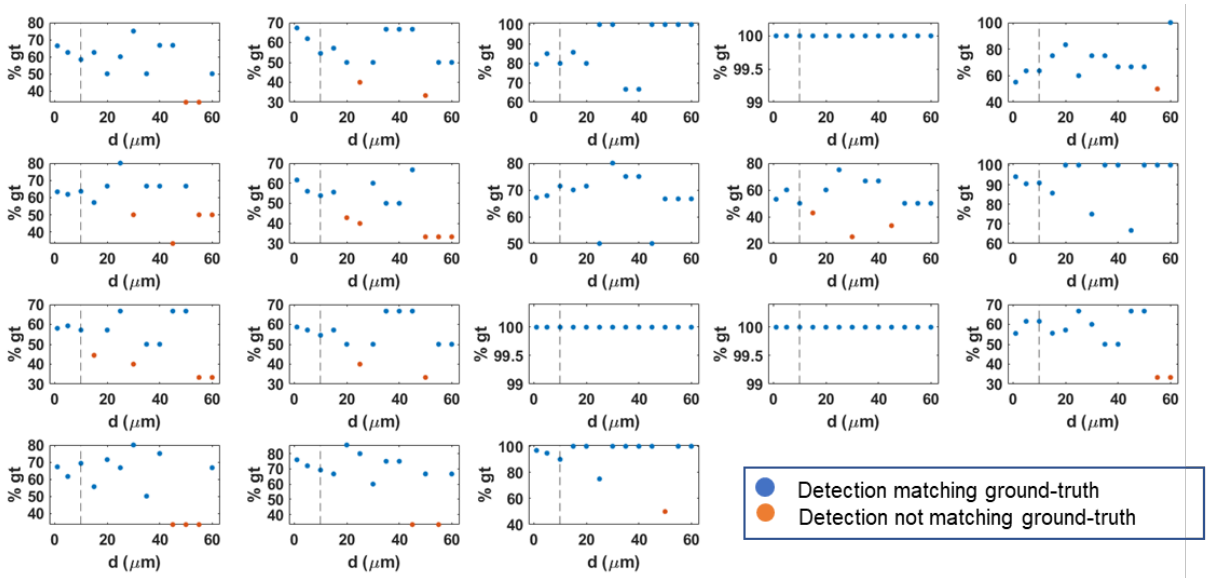

Supplementary Figure 12. IBM evaluation of sparse predictions for maximum z-slice interval: Each plot represents one live embryo instance. z-slices for each embryo were selected according to the increasing interval between them starting from 1 µm to 60 µm (x-axis of each plot). Correspondingly, the % of the detected class is plotted on the y-axis. The black vertical line on each graph shows the maximum interval (10 µm) after which error starts to appear. Blue dots are correct predictions (matching to the ground truth) and an orange dot appears when the model flips the prediction to the wrong class due to insufficient z-slices.

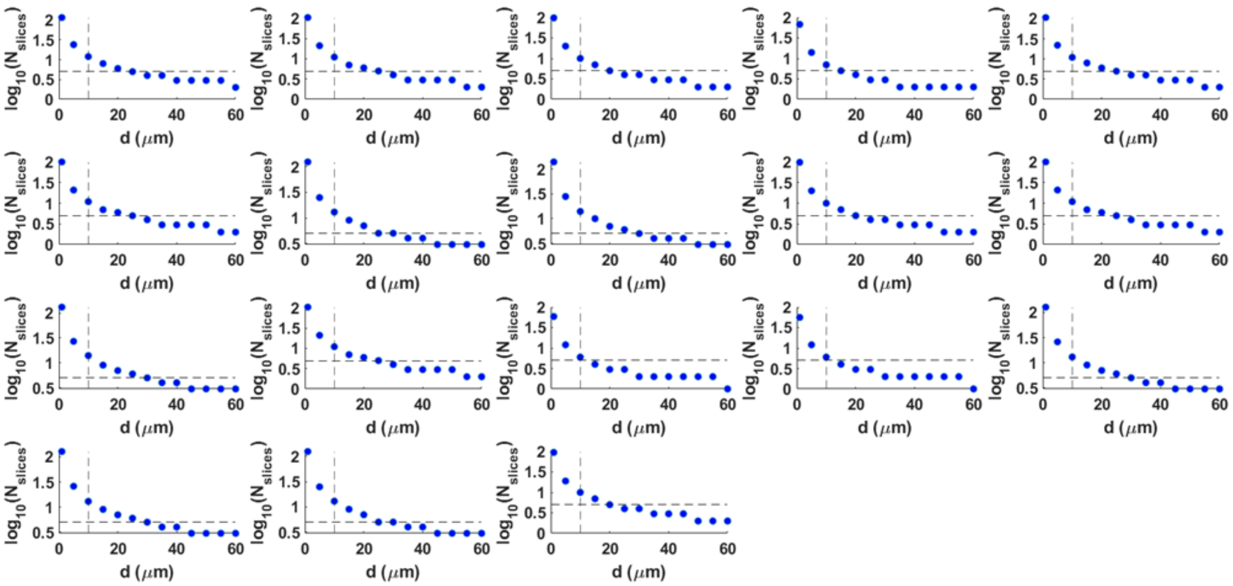

Supplementary Figure 13. IBM evaluation of sparse predictions for minimum number of z-slices: Each plot represents one live embryo instance. z-slices for each embryo were selected according to the increasing interval between them starting from 1 μm to 60 μm (x-axis of each plot). The corresponding number of z-slices to cover the entire embryo are shown on the y-axis in log10 scale. Black vertical line is the maximum interval (10 μm) determined from Supplementary Fig. 12. For intervals less than 10 μm, the number of z-slices are greater than 5, shown by the dotted black horizontal line.

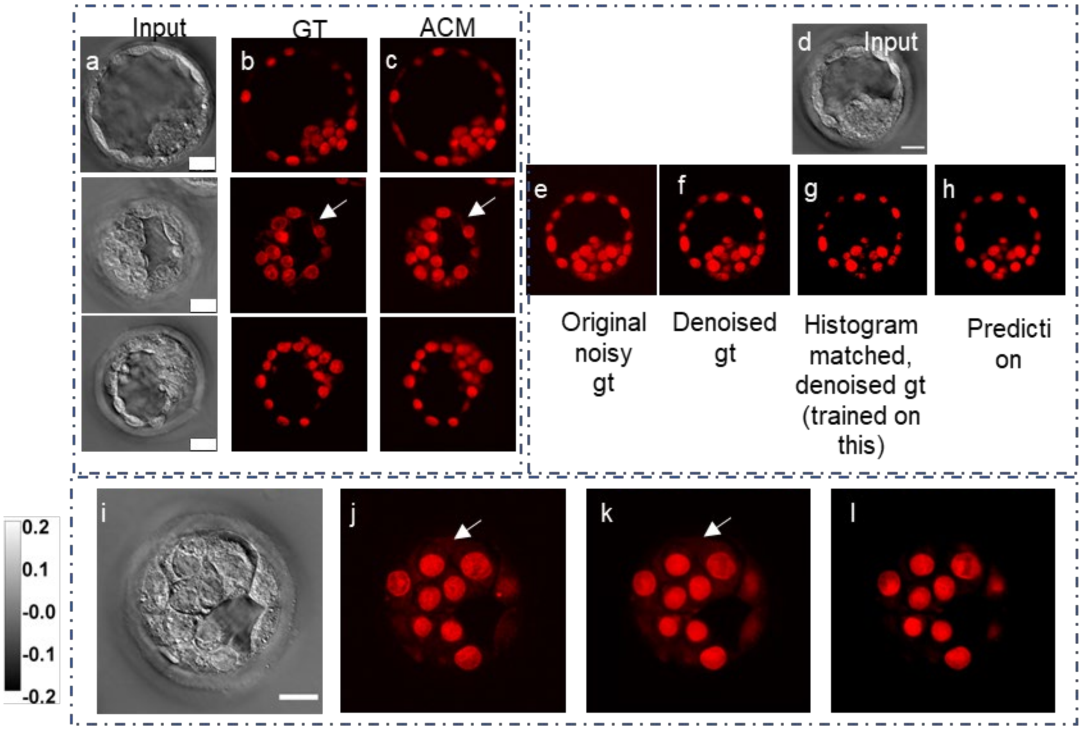

PSNR	MSSSIM	PCC
35.08	0.92	0.92

PSNR	MSSSIM	PCC
36.94	0.94	0.81

Supplementary Figure 14. Nucleus prediction model preprocessing: a. Input: LS-GLIM , b. ground truth fluorescence, and c. model predictions without histogram matching. Preprocessing for the final model: d. input LS-GLIM, e. original ground truth, f. denoised ground truth, g. histogram matched to input ground truth, and h. the final prediction by training on images like (d, g) pair. i. one LS-GLIM image, j. Real denoised fluorescence ground truth, k. Non-histogram-matched model predictions, l. Predictions by final model trained on histogram matched denoised ground truth. White arrows indicate spurious cytoplasm signals. Scalebar indicated by white rectangle on lower right corner of each LS-GLIM image is 20 µm and also applies to the corresponding fluorescence and model prediction images.

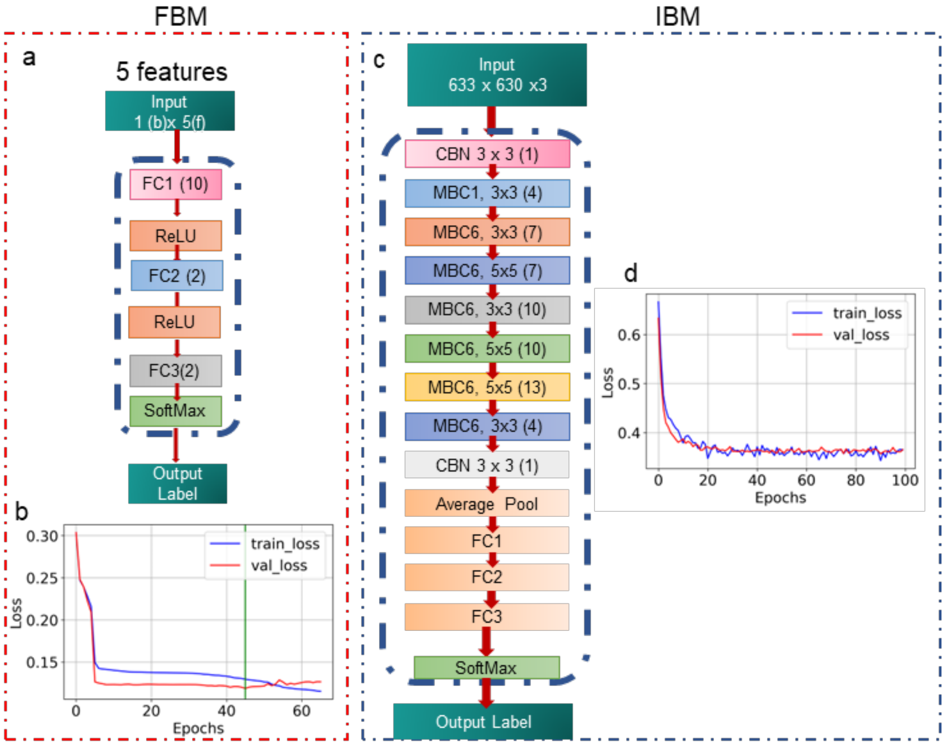

Supplementary Figure 15. Health grading networks architecture: a. FBM architecture- a three layered neural network classifier with hidden units [10, 2] b. Loss curve for FBM with a vertical green line denoting the chosen model. c. IBM architecture-an Efficient Net B7. The classifier layer of the model is replaced with three fully connected layers for 2-class classification Corresponding loss curve is shown in d. CBN: Conv2d, batch normalization, activation, MBC: MBConv Block, FC: Fully connected layers.

Supplementary Note 1: LS-GLIM setup

Light from the laser source is directed through the beam steering components inside the confocal module and scanned through the transverse plane of the sample using scanning mirrors SM1 and SM2. The microscope is configured in a differential interference contrast (DIC) mode with a Nomarski prism (NP) beneath the objective that divides the incoming light beam into two laterally sheared orthogonal polarizations. These laterally sheared orthogonal polarization beams travel through the sample and are collected by a condenser lens (CL). After passing through a second Nomarski prism in the condenser, the two laterally sheared beams are combined into a single beam. This composite beam now enters the GLIM module that comprises a liquid crystal variable retarder (LCVR) to introduce modulations in phase. The LCVR provides four phase shifts in steps of $\pi/2$ between the two orthogonal polarization components of the beam. An analyzer (A) placed at 45 deg to either polarization enables interference which is detected by the transmission photomultiplier tube (T-PMT). After recording four intensity images at the T-PMT, a phase-shifting reconstruction algorithm is applied to extract the phase gradient[1,2]. The final phase image, shown in Fig. 1b, is obtained by integrating the phase gradient image along the shear direction[2]. The corresponding fluorescence image (Fig. 1c) for the same field of view is captured through the confocal detector (CD) in the epi-illumination light path after passing through a pinhole (P) and an appropriate emission filter on the emission filter wheel (EFW) as shown in Fig. 1a.

Supplementary Note 2: MATLAB app operation

The app is a demonstration of our workflow for segmentation of nucleus prediction images in 2D as well as 3D, visualization and analysis of the nuclei/embryo descriptor features and prediction of the health class of an embryo using the trained FBM or IBM. For testing, we provide datasets for two embryos, one in H/I class and the other in S class. There are three panels in the app: First panel titled ‘Segment’ is for the segmentation of the nucleus prediction results. The second panel titled ‘Analyze’ is for feature extraction and health grading using FBM or IBM. The third panel is titled ‘Analyze on few slices’, this panel is for health grading of live embryos using sparse predictions by IBM.

We will now discuss each of these panels in detail:

Segment Panel

The layout of this panel is shown in Supplementary Fig. 16.

This panel is for 2D (Supplementary Fig. 16a) and 3D (Supplementary Fig. 16b) segmentation of the nucleus predictions of an embryo.

Required inputs in Supplementary Fig. 16 are:

1. **pth**: is the system path of the main folder, with the required subfolders to be named as
   a. **glim**: for storing raw GLIM images from LS-GLIM acquisition. This folder is to be generated by the user.
   b. **cropped**: for storing 1280 x 1280 sized cropped images from glim folder. This folder is to be generated by the user.
   c. **new**: for storing nucleus prediction images of cropped folder images. This folder is to be generated by the user and the nucleus prediction is performed outside the MATLAB environment.
   d. **overlapped**: for storing 3-channel z-slice images for IBM predictions. This folder is generated by the app.
   e. **2d_3d_segmentation_final**: for storing segmentation data with subfolders: **'label3'** (2D labels of 3D volume), **'seg_3d_3'** (3D labels of 3D volume), **'seg_centroid3'** (centroid distribution of downsized volume) and **'data_resize'** (extracted 3D features).
2. **med_filter_window:** size of the initial 2D median filter, the filter will be applied to each 2D image.
3. **hard_threshold:** First threshold after normalization of 2D image.
4. **sensitivity:** sensitivity of adapthresh MATLAB function.
5. **adapt_wind:** Neighborhood size parameter of adapthresh MATLAB function.
6. **h:** connectivity parameter of imextendedmin MATLAB function, can be 4 or 8.
7. **solidity:** the solidity cut-off of the detected nuclei objects.
8. **image_size:** size of the square input image.
9. **min_debris pixels:** maximum size of discarded objects before the watershed operation in pixels.

10. **min_area pixels:** Objects with area less than the min_area will be discarded after the watershed operation. Objects with area >= 1.5 times min_area and solidity greater than solidity cut-off will be selected for final mask. pixel_ratio: pixels per μm for the xy dimension.
11. **objective_NA:** numerical aperture of the objective lens used for imaging.
12. **imaging _z_step:** step size of the z-scan in μm.
13. **lambda:** wavelength for LS-GLIM acquisition in μm.
14. **Volume_gating:** Maximum volume cut-off to remove undersegmented nucleus in μm^3.
15. **gap_z:** Maximum allowed gaps in trajectory in z-direction
16. **nuc_radius:** parameter for determining trajectory of centroid in z-direction.
17. **sensitivity:** sensitivity*nuc_radius determines the amount in pixels in lateral dimensions to search for the next centroid in the adjacent z-sections.
18. **slices to exclude:** use this parameter if the first and last z-sections of the acquired stack do not contain in-focus information. Processing will then be applied to the rest of the z-sections after excluding the specified number of z-sections from top and bottom of the stack.

The test button runs the 2D segmentation on one randomly selected z-section nuclei prediction image and displays the raw image and the corresponding label map in figure panels marked as Supplementary Fig. 16 c, d respectively. run all button runs the 2D segmentation on whole data and saves the corresponding labels in *pth→2d_3d_segmentation_final→label3* folder.

For the 3D segmentation, test3D runs the 3D segmentation and shows the 3D predicted nuclei volume and the corresponding label volume in Supplementary Fig. 16e and f respectively without storing intermediate outputs. This feature is useful for a quicker assessment of 3D segmentation parameters as compared to a full run. Run button performs the 3D segmentation and writes the 3D labels in *pth→2d_3d_segmentation_final→seg_3d_3* and the resized centroid volume in *pth→2d_3d_segmentation_final→ seg_centroid3* folders respectively. The show 3D labels button displays the already stored 3D predicted nuclei volume and the corresponding label volume in Supplementary Fig. 16e and f respectively.

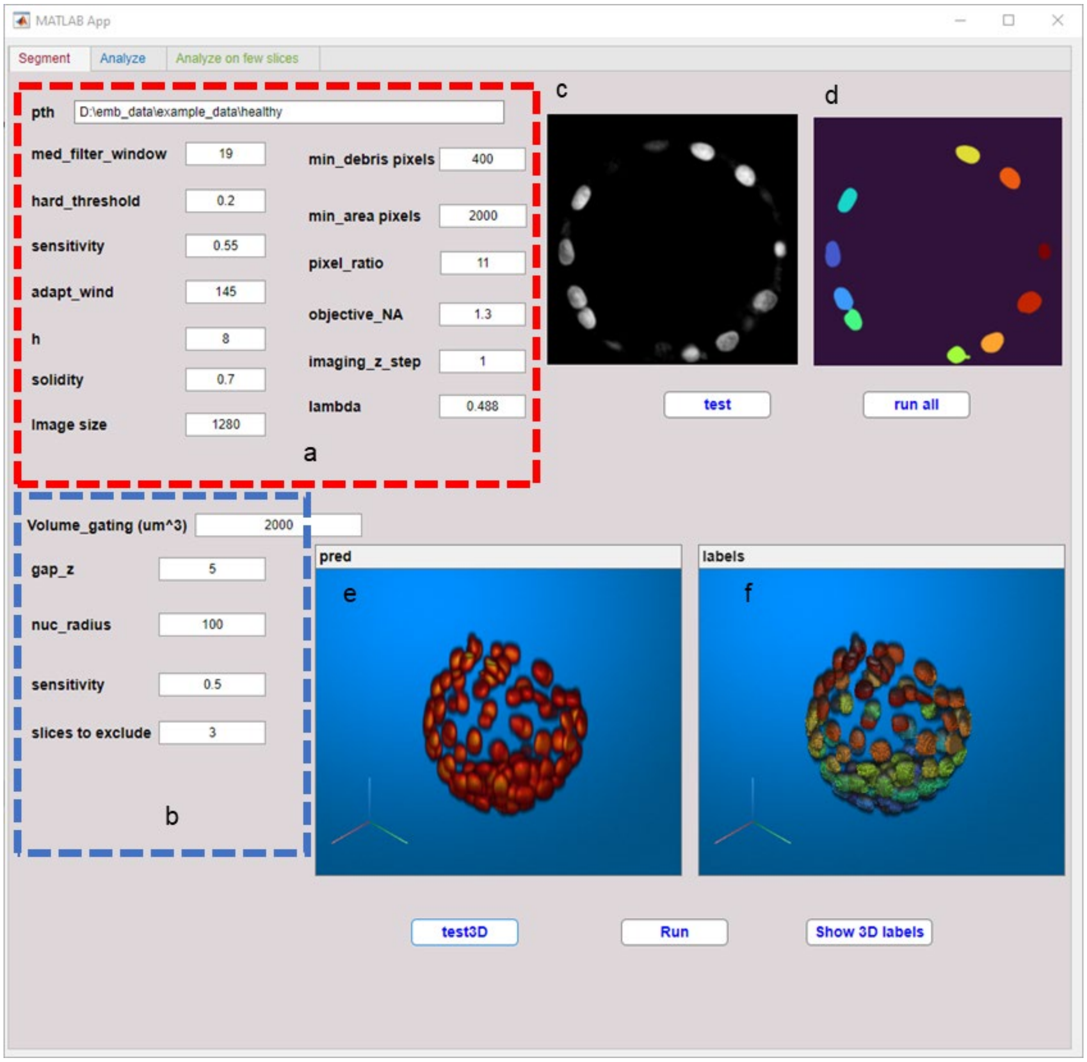

Supplementary Figure 16. MATLAB app panel 1: Segment. User defined parameters for a. 2D segmentation b. 3D segmentation c. One z-section of nucleus prediction d. Corresponding generated 2D nuclei instance segmentation label e. 3D stacked nuclei prediction f. 3D nuclei instance segmentation map with each nucleus represented by different colors.

Analyze Panel

This panel is used to extract 3D features from the segmented nuclei volume of an embryo and perform its health classification. Required inputs in Supplementary Fig. 17a include:

1. **base_path:** same as **pth** parameter in Supplementary Fig. 16a.
2. **pth_1d_model:** system path to the FBM (trained in MATLAB).
3. **model_name:** name of the trained FBM model mat file.

The rest of the inputs are the same as in Supplementary Fig. 16 explained previously. The Exclude slices input is for IBM predictions.

Show composite glim and nucleus prediction button shows the maximum value projection along z-direction of the LS-GLIM and the corresponding nucleus prediction volume (Supplementary Fig. 17b). Show 3D labels button displays the nuclei prediction volume and the corresponding label volume (Figs. S17c and d respectively) and also displays the number of nuclei detected in the embryo. Extract parameters and predict-feature model button performs the 3D feature measurements of the labelled volume and classifies the health of the embryo using trained FBM. Show Nuclear dry mass density map button displays the mean nuclear dry mass density distribution map (Supplementary Fig. 17e) with the colorbar below indicating the values in pg/μm^3. Based on the FBM output, green LED lights up against H/I-feature text if the embryo is predicted to be of H/I class or a red LED lights up against the S-feature text if the embryo is predicted to be of S class, with the percentage of nuclei in favor of the predicted health class denoted by the slider (Supplementary Fig. 17f). The histograms of all calculated features are displayed in Supplementary Fig. 17g. Prepare 3-slice data prepares 3-channel z-slice data from the **cropped** folder and writes the data in the **overlapped** folder. IBM predictions are performed outside MATLAB environment. IBM outputs a **check.csv** file that is saved in the **overlapped** folder in the main base folder (**pth**). Predict-Image-model performs the max-voting on individual z-slice predictions of IBM from the check.csv file and the health classification and the percentage of z-slices in the favor of predicted class are displayed in Supplementary Fig. 17h in a manner similar to Supplementary Fig. 17f.

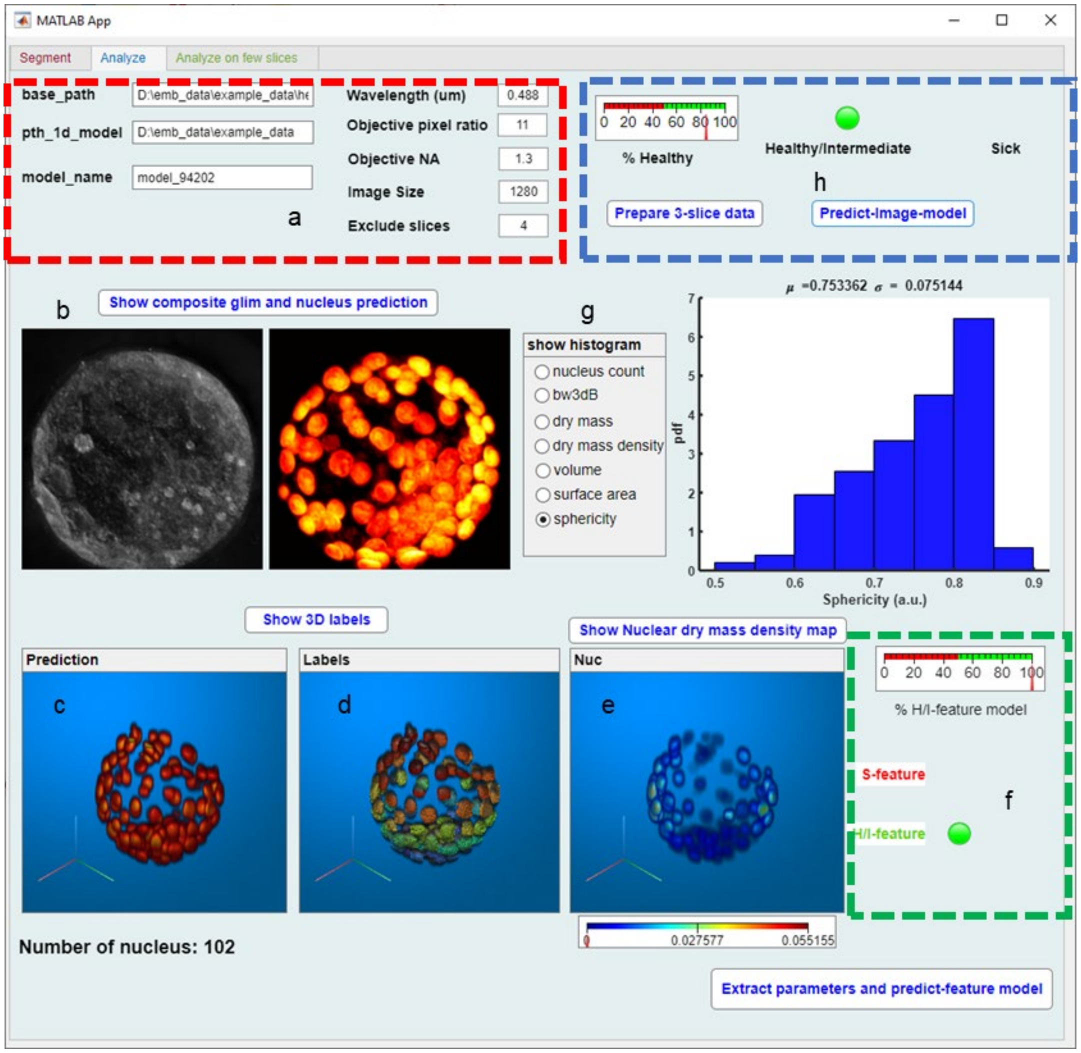

Supplementary Figure 17. MATLAB app panel 2: Analyze. a. User input, b. Composite LS-GLIM and nucleus prediction c. 3D stacked nucleus prediction d. 3D segmented labels, e. Mean dry mass density map per nucleus, f. Health grading by FBM, g. Histograms of extracted features, h. Health grading by IBM.

Analyze on few slices Panel

This panel is a demo of sparse predictions feature of the IBM on live embryo data. Inputs in this panel (Supplementary Fig. 18a) are:

1. **pth_to_base:** system path to the live embryo data
2. **prediction_filename:** IBM output csv file name

3. **ground_truth_filename:** name of the file containing z-slice names from which z position is extracted.
4. **glim folder:** folder containing 3 channel z-slice images to show the composite images (Supplementary Fig. 18b)

Show composite button shows a montage of z-slice images of an embryo in steps of 3 µm. The z-slices positions are mentioned on the top of every tile (Supplementary Fig. 18b). Random selections of z-slice positions can be made through the check box panel (Supplementary Fig. 18c). When the required number of z-slice positions are selected (Supplementary Fig. 18d), the Done switch should be turned 'On' to enable embryo-level prediction. The result is shown against the Status text and the percentage of z-slices in favor of the predicted class is displayed on the slider (Supplementary Fig. 18e). To restart the process, the restart switch should be turned to On position.

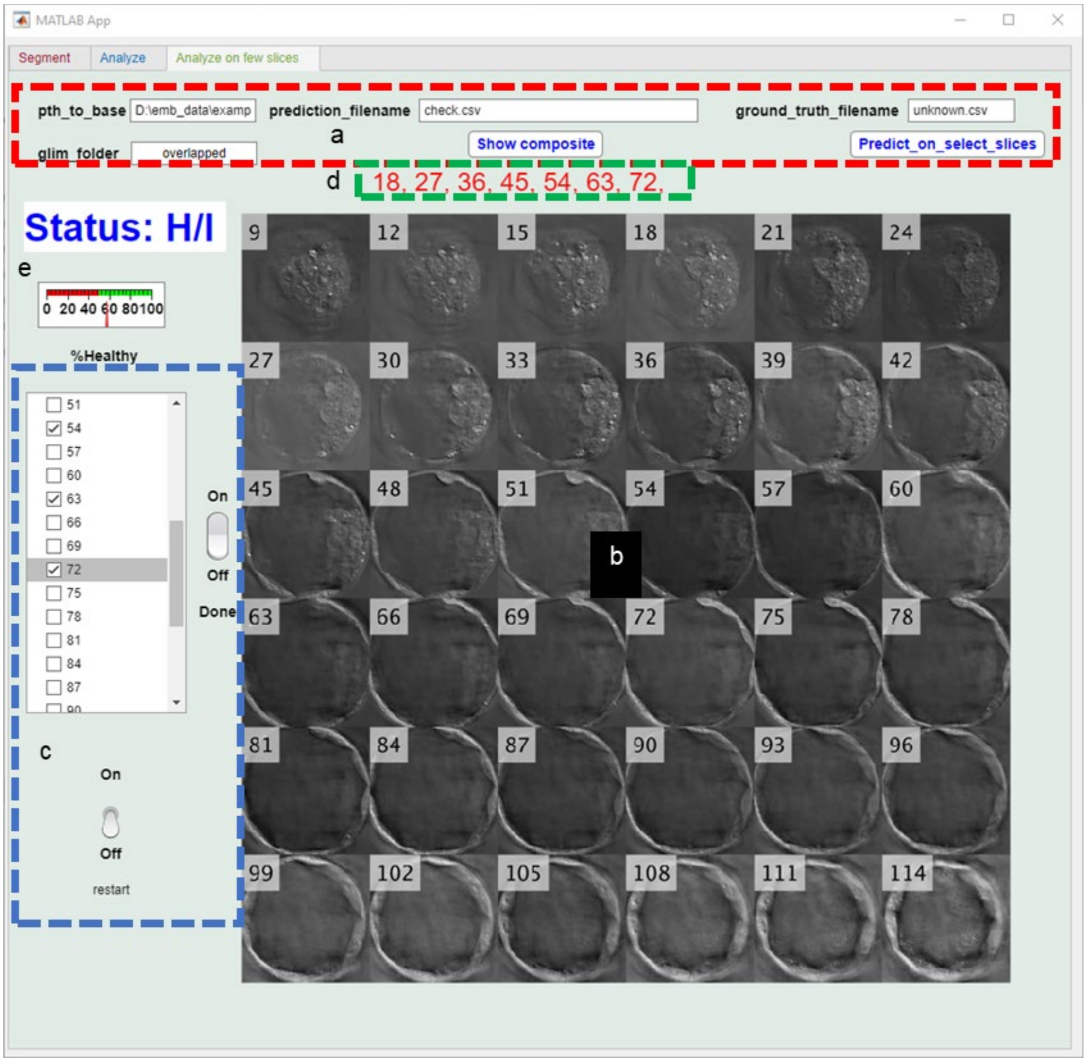

Supplementary Figure 18. MATLAB app panel 3: Analyze on fewer slices: a. User input, b. Montage of z-slices of the individual embryo, c. Slice selection menu d. Selected z-slices, e. Health status by IBM on selected slices.

Feature	P-val (Normality) Lilliefors Test (alpha=0.05)			KW-statistics (alpha=1e-3)	P-val (Dunn-Multiple comparison) (alpha=1e-3)			Sample-size
	H	I	S	P-val	HI	IS	HS	[H,I,S]
dm	0.0009	0.0009	0.0009	0.79e-54	0.45	0.11e-50	0.23e-43	[2775,3827, 1186]
dmd	0.0009	0.0009	0.0009	0.82e-73	0.23	0.18e-63	0.22e-64	[2775,3827, 1186]
volume	0.0009	0.0009	0.0009	0.002	0.012	0.012	0.544	[2775,3827, 1186]
surface_area	0.0009	0.0009	0.0009	0.19e-5	0.1	0.88e-6	0.38e-3	[2775,3827, 1186]
sphericity	0.0009	0.0009	0.0009	0.46e-19	0.55e-4	0.18e-10	0.12e-19	[2775,3827, 1186]
Nuc_count	0.69	0.17	0.77	0.36e-14	0.76	0.60e-12	0.27e-11	[46,65,41]
bw3dB	0.102	0.49	0.53	0.25e-15	0.82	0.51e-13	0.47e-12	[46,65,41]

Feature	Levene test P-val	One-way ANOVA P-val	P-val (post-hoc Student T test) (alpha=1e-3)		
			HI	IS	HS
Nuc_count	0.399	0.14e-20	0.63	0.17e-17	0.25e-14
bw3dB	0.002	0.17e-21	0.82	0.63e-18	0.22e-17

Supplementary Table 1. Statistical test results

name	actual_class	IBM_prediction	FBM_prediction
h4_d10_f2	H/I	H/I	H/I
h4_d1_f2	H/I	H/I	H/I
h4_d1_f4	H/I	H/I	H/I
h4_d5_f2	H/I	H/I	H/I
h4_d7_f2	H/I	H/I	H/I
h4_d8_f3	H/I	H/I	H/I
h4_d8_f4	H/I	H/I	H/I
h4_d9_f1	H/I	H/I	H/I
h5_d10_f3	H/I	H/I	H/I
h5_d11_f4	H/I	H/I	H/I
h5_d2_f1	H/I	H/I	H/I
h5_d5_f1	H/I	H/I	H/I
h5_d5_f2	H/I	H/I	H/I
h5_d6_f1	H/I	H/I	H/I
h5_d6_f2	H/I	H/I	H/I
h5_d6_f3	H/I	H/I	H/I
h5_d7_f1	H/I	H/I	H/I
h5_d8_f1	H/I	H/I	H/I
h1_d2_f1	H/I	H/I	H/I
h1_d2_f3	H/I	H/I	H/I
h1_d3_f5	H/I	S	H/I
h2_d2_f1	H/I	H/I	H/I
h2_d3_f1	H/I	H/I	H/I
h2_d3_f10	H/I	H/I	H/I
h2_d3_f4	H/I	H/I	H/I
h2_d5_f2	H/I	H/I	H/I
h2_d5_f5	H/I	H/I	H/I
h2_d5_f7	H/I	H/I	H/I
h2_d5_f9	H/I	H/I	H/I
h3_d2_f2	H/I	H/I	H/I
h3_d6_f1	H/I	H/I	H/I
s4_d10_f8	S	S	S
s4_d1_f5	S	S	S
s4_d1_f7	S	S	S
s4_d9_f3	S	S	S
s5_d11_f1	H/I	H/I	H/I
s5_d1_f1	S	S	S
s5_d4_f5	S	S	S
s5_d6_f7	S	S	S
s5_d8_f2	S	S	S
s5_d8_f5	S	S	S
s5b_d10_f1	S	S	S
s5b_d11_f1	S	S	S
s5b_d12_f1	S	S	S
s5b_d13_f1	S	S	S
s5b_d14_f1	S	S	S
s5b_d1_f1	S	S	S
s5b_d2_f1	S	S	S
s5b_d3_f1	S	S	S
s5b_d4_f1	S	S	S
s5b_d5_f1	S	S	S
s5b_d6_f1	S	S	S
s5b_d7_f1	S	S	S
s5b_d8_f1	S	S	S
s5b_d9_f1	S	S	S
s1_d1_f5	H/I	H/I	H/I
s1_d2_f2	S	S	S
s1_d4_f1	S	S	S
s1_d5_f2	S	S	S
s2_d1_f2	H/I	H/I	H/I
s2_d1_f4	H/I	H/I	H/I
s2_d2_f1	H/I	H/I	H/I
s2_d2_f4	H/I	H/I	H/I
s2_d3_f8	H/I	H/I	H/I
s2_d4_f4	H/I	H/I	H/I
s3_d1_f3	H/I	H/I	H/I
s3_d3_f1	H/I	H/I	H/I
s3_d5_f2	S	S	S
s3_d5_f4	H/I	H/I	H/I
s3_d6_f4	H/I	H/I	H/I
s3_d7_f1	H/I	H/I	H/I
s3_d7_f3	H/I	H/I	H/I

Supplementary Table 2. Test performance on common in-distribution data of 72 fixed embryos.

name	actual_class-exp1	actual_class-exp2	IBM	FBM
211	H/I	H/I	H/I	H/I
212	H/I	H/I	H/I	H/I
213	H/I	H/I	H/I	H/I
221	H/I	S	S	H/I
222	H/I	H/I	S	H/I
223	S	S	S	S
311	H/I	H/I	H/I	S
312	H/I	H/I	H/I	H/I
313	S	S	S	S
314	S	S	S	S
321	H/I	H/I	H/I	H/I
322	H/I	H/I	H/I	H/I
323	H/I	H/I	H/I	H/I
324	S	S	S	S
111	H/I	H/I	H/I	H/I
121	H/I	H/I	H/I	S
122	H/I	I/S	S	S
123	S	S	S	S
124	S	S	S	S
131	H/I	S	S	S
141	S	S	S	S
161	S	S	S	S
		ACCURACY (Excluding expert decision mismatch)	0.947	0.895

Supplementary Table 3. Test performance on out-of-distribution data of 19 time-instances of 8 embryos

Model Number	Model Type	Model Information	Status	Accuracy % (Test)	Accuracy % (Live)
2.29	Neural Network	Bilayered [10,10]	Tested	98.17	60.39
2.7	Naive Bayes	Gaussian Naïve Bayesian	Tested	93.01	58.13
2.27	Neural Network	Medium [25]	Tested	98.12	56.73
2.15	KNN	Fine KNN	Tested	97.44	55.76
2.28	Neural Network	Wide 100	Tested	97.86	54.68
2.4	Discriminant	Linear	Tested	97.86	54.68
2.26	Neural Network	Narrow 10	Tested	98.54	54.14
2.3	Neural Network	Trilayered [10,10,10]	Tested	99.32	53.82
2.9	SVM	Linear	Tested	99.32	53.71
2.6	Logistic Regression	Logistic	Tested	98.54	53.07
2.11	SVM	Cubic	Tested	99.06	51.45
2.2	KNN	Weighted	Tested	98.23	50.91
2.5	Discriminant	Quadratic	Tested	96.56	50.91
2.8	Naive Bayes	Kernel-Gaussian	Tested	95.04	50.81
2.13	SVM	Medium Gaussian	Tested	99.69	50.38
2.12	SVM	Fine Gaussian	Tested	97.60	49.84
2.23	Ensemble	Subspace Discriminant	Tested	98.43	49.84
2.18	KNN	Cosine	Tested	98.33	49.84
2.14	SVM	Coarse Gaussian	Tested	99.63	48.98
2.3	Tree	Coarse Tree	Tested	98.17	48.87
2.16	KNN	Medium	Tested	98.80	48.87
2.17	KNN	Coarse	Tested	99.79	48.55
2.19	KNN	Cubic	Tested	98.75	48.33
2.32	Kernel	Logistic regression kernel	Tested	94.73	48.22
2.24	Ensemble	Subspace KNN	Tested	95.67	47.47
2.1	Tree	Fine Tree	Tested	97.03	46.18
2.2	Tree	Medium Tree	Tested	100.00	46.18
2.25	Ensemble	RUSBoosted Tree	Tested	98.17	46.18
2.21	Ensemble	Adaboosted Tree	Tested	100.00	46.18
2.1	SVM	Quadratic	Tested	99.84	44.67
2.31	Kernel	SVM Kernel	Tested	97.60	43.92
2.22	Ensemble	Bagged Trees	Tested	98.17	40.37

Supplementary Table 4. Initial model selection results-MATLAB

Supplementary References:

1 Creath, K. Phase-measurement interferometry techniques. *Progress in optics* **26**, 349-393 (1988).

2 Nguyen, T. H., Kandel, M. E., Rubessa, M., Wheeler, M. B. & Popescu, G. Gradient light interference microscopy for 3D imaging of unlabeled specimens. *Nature communications* **8**, 1-9 (2017).
