# Supplementary material for: EVATOM: an optical, label-free, machine learning assisted embryo health assessment tool
Source: Commun Biol. 2024 Mar 5;7:268. doi: 10.1038/s42003-024-05960-w (PMC10915136; doi:10.1038/s42003-024-05960-w)
Supplement: Supplementary file 2 — Description of Supplementary Materials [file 42003_2024_5960_MOESM2_ESM.pdf]

Description of Additional Supplementary Files

File name: Supplementary Data 1
Description: Source data for graphs and box plots in the manuscript.

File name: Supplementary Movie M1
Description: Movie showing a z-scan of an embryo with overlay of LS-GLIM (gray) and nucleus predictions by NPM (red).

File name: Supplementary Movie M2
Description: Movie illustrating the 3d visualization of stacked ground truth (fluorescence) and stacked nuclei predictions on a test embryo for comparison.

File name: Supplementary Movie M3
Description: Movie depicting 3D renderings of LS-GLIM, nuclei prediction, segmentation labels, and mean nuclear dry mass density map for an embryo.

File name: Supplementary Movie M4
Description: Movie demonstrating the MATLAB app operation for EVATOM.
